# Positive and Negative Thinking in Tinnitus: Factor Structure of the Tinnitus Cognitions Questionnaire

**DOI:** 10.1097/AUD.0000000000000365

**Published:** 2016-12-19

**Authors:** Lucy E. Handscomb, Deborah A. Hall, Gillian W. Shorter, Derek J. Hoare

**Affiliations:** 1 Division of Clinical Neuroscience, National Institute of Health Research Nottingham Hearing Biomedical Research Unit, Otology and Hearing Group, Nottingham, United Kingdom; 2 University College London Ear Institute, London, United Kingdom; and 3 Alcohol and Public Health Team, Teesside University, United Kingdom.

**Keywords:** Cognition, Thinking, Tinnitus, Questionnaire

## Abstract

**Objectives:**

Researchers and clinicians consider thinking to be important in the development and maintenance of tinnitus distress, and altering thoughts or thinking style is an object of many forms of psychological therapy for tinnitus. Those working with people with tinnitus require a reliable, psychometrically robust means of measuring both positive and negative thinking related to it. The Tinnitus Cognitions Questionnaire (TCQ) was designed as such a measure and its authors showed it to be reliable, with good psychometric properties. However, no research teams have yet carried out independent validation. This study aimed to use the TCQ to investigate thinking amongst members of the general population with both bothersome and nonbothersome tinnitus and also to verify its factor structure.

**Design:**

Three hundred forty-two members of the public with tinnitus completed the TCQ online or on paper. They also rated their tinnitus on a scale as “not a problem,” “a small problem,” “a moderate problem,” “a big problem,” or a “very big problem.” The authors tested the original factor structure of the TCQ using confirmatory factor analysis and then calculated the mean scores for each item, comparing mean total scores across “problem categories” for the full questionnaire and for the positive and negative subscales.

**Results:**

The original two-factor structure of the TCQ was a good fit to the data when the correlation between positive and negative factors was fixed at zero (root mean square error of approximation = 0.064, 90% confidence interval = 0.058 to 0.070). Items pertaining to wishing the tinnitus would go away and despairing that it would ever get better had the highest mean scores. The mean total score for the “no problem” group (M = 31.17, SD = 16.03) was not significantly different from the mean total score for the “small problem” group (M = 34.00, SD = 12.44, *p* = 0.99). Differences between mean scores for all other groups were statistically significant. For the negative subscale, differences were statistically significant between all problem categories. For the positive subscale, the differences between mean scores were only statistically significant for the “no problem” group (M = 28.40, SD = 17.11) compared with the “moderate problem” group (M = 18.55, SD = 8.64, *p* = 0.02) and for the “moderate problem” group compared with the “very big problem” group (M = 26.79, SD = 11.66, *p* = 0.002). Positive and negative factors were uncorrelated (*ρ* = −0.03.)

**Conclusions:**

The TCQ is a valid measure of positive and negative thinking in tinnitus, and the authors recommend its use in research and therapeutic settings. Negative thinking appears to be associated with more problematic tinnitus, but positive thinking is not associated with unproblematic tinnitus, suggesting that reducing negative thinking may be more important than teaching positive thinking in therapy.

## INTRODUCTION

Tinnitus is experienced by around 10% of the UK population, but only about half of these people describe it as moderately or severely annoying (Davis & El Refaie 2000). Understanding why tinnitus is more annoying or distressing to some people than others has long been an important focus of research (Fowler 1942; Hiller & Goebel 2007). An early psychological perspective on tinnitus distress based on clinical observations (Hallam et al. 1984) noted that many people habituate to tinnitus naturally, so that over time it becomes unremarkable in the same way as the sound of distant traffic. However, some people do not habituate to the tinnitus sound, and Hallam et al. (1984) suggested that negative thoughts about the significance of tinnitus or one’s ability to tolerate it may be one factor that prevents habituation. Zenner and Zalaman (2004) expressed similar ideas, suggesting that cognitive processes cause sensitization to tinnitus in some individuals. The cognitive model of tinnitus distress (McKenna et al. 2014) places great importance on negative thinking. It suggests when someone engages in negative thinking about tinnitus and its meaning, this triggers a cycle of emotional distress, selective attention, and avoidance behavior, which ensures that tinnitus remains a distressing experience. Budd and Pugh (1996) postulated that catastrophizing (overly negative thinking) is a key component of a maladaptive coping style, which they found to be associated with greater emotional distress. Empirical evidence for an association between catastrophic thinking and tinnitus distress in clinical populations has more recently been provided by several cross-sectional questionnaire studies (Cima et al. 2011; Weise et al. 2013; Conrad et al. 2015b).

A question that has so far been little investigated is the influence of thinking positively (as opposed to not thinking negatively) on people’s experience of tinnitus. Wilson and Henry (1998) propose that “constructive” thoughts about tinnitus, such as “it generally gets better after a while” protect people from emotional distress. However, contrary to their expectations, Budd and Pugh (1996) found that “effective coping,” characterized by positive self-talk (such as reassuring oneself that it is possible to cope with tinnitus) did not correlate with lower levels of tinnitus distress, whereas the absence of maladaptive coping did.

Altering cognitions or thoughts related to tinnitus is a core component of several forms of tinnitus therapy. Cognitive behaviour therapy (CBT), which includes learning how to identify and modify negative thoughts, has been shown in several studies to be effective in reducing tinnitus distress (for reviews, see Hesser et al. 2011; Hoare et al. 2011). Although an important part of psychologically based therapy for tinnitus is the identification and modification of negative thoughts, it contains other important components as well, such as behavioral activation, applied relaxation, and problem-solving. Measuring thoughts with a questionnaire designed explicitly for that purpose helps to establish whether improvements are due to changes in thinking or some other effect, but not all investigations have done this. Those studies which have measured thinking separately from tinnitus distress using a questionnaire or subscale (Henry & Wilson 1996; Kroner-Herwig et al. 2003; Hiller & Haerkotter 2005; Cima et al. 2012; Conrad et al. 2015a) have all shown that thinking does alter after CBT-based intervention. Henry and Wilson (1996) described a “cognitive restructuring” technique, which also involves the deliberate modification of negative thoughts. For example, “I’ll never have peace and quiet again” might be modified to “I can feel at peace through relaxation even though things won’t be totally quiet.” A study group who learned this technique along with attention-switching (shifting attention from tinnitus onto a part of the body and back again) and mental imagery exercises (e.g., visualizing tinnitus as a waterfall) experienced greater improvements in tinnitus distress than a group who received education alone. Scores on the Tinnitus Cognitions Questionnaire (TCQ; Wilson & Henry 1998) as well as Tinnitus Reaction Questionnaire (Wilson et al. 1991) reduced, which suggests that changes in thinking contributed to a lessening of tinnitus-related emotional distress. However, scores for negative and positive thinking subscales of the TCQ were not reported separately.

In recent years, there has been increasing interest in acceptance and commitment therapy (ACT; Hayes et al. 2011) and mindfulness-based CBT (Segal et al. 2002) as alternative means of reducing tinnitus distress. In both of these approaches, the focus is not on changing thoughts but on learning to disengage with them so that they have less influence on feelings (Williams et al. 2007; Hayes et al. 2011). ACT has been shown to be effective in reducing tinnitus distress when delivered both face-to-face (Westin et al. 2011) and over the internet (Hesser et al. 2014), although the latter study did not find it to be more effective than conventional CBT. Only preliminary mindfulness studies have been conducted so far, but early results are promising (Sadlier et al. 2008; Philippot et al. 2012; Gans et al. 2014). Given that ACT teaches acceptance of one’s thoughts as they are and mindfulness meditation shifts the emphasis away from learning to think more positively toward simply observing one’s thoughts, the relative importance of thinking positively versus not thinking negatively has become a priority for investigation. A reliable, psychometrically robust means of measuring both positive and negative thinking related to tinnitus is therefore important.

To fully test the proposed models of tinnitus distress, thinking needs to be measured separately from other possible contributing factors such as selective attention or avoidance behavior. To thoroughly evaluate the effectiveness of psychological therapy, changes in positive and negative thinking need to be assessed in addition to overall tinnitus-related emotional distress. The TCQ was developed in 1998 to measure the content and frequency of positive and negative thoughts related to tinnitus. Other measures of thinking in tinnitus have been developed since. Catastrophizing can be measured by the Tinnitus Catastrophizing Scale (Cima et al. 2011) or the Tinnitus-Related Self-Statements Scale (Flor & Schwarz 2003). The Tinnitus Cognitions Scale (Conrad et al. 2015b) is designed to measure both catastrophizing and thoughts about avoidance. All three of these questionnaires focus on negative thinking. The TCQ is unique in measuring both positive and negative thoughts.

The TCQ was first reported in a clinical trial (Henry & Wilson 1996) and subsequently a validation study (Wilson & Henry 1998). It is derived from an earlier, unpublished questionnaire that consisted of 20 items chosen on the basis of interviews with patients about their thoughts concerning their tinnitus (no further detail about item selection is provided). The unpublished version presented positive and negative items in random order but, as responders found this confusing, it was restructured so that items pertaining to negative and positive thoughts were grouped together.

A psychometrically robust questionnaire has considerable potential to collect important information about thinking related to tinnitus, to measure the effectiveness of psychological therapies in both clinical and research settings, and to represent the experience of those with tinnitus. To date, no independent validation of the TCQ has been carried out beyond the original study by Wilson & Henry (1998). As such, it is important to determine if the two factor structure holds, using the more parsimonious confirmatory factor analysis (CFA) and accounting for the categorical nature of the data. The present study used data from a sample of people with tinnitus from the general UK population (1) to investigate the psychometric properties of the TCQ, in particular if the factor structure proposed by Wilson and Henry holds, and if the subscales are reliable and (2) to determine if scores on these factors differ between groups of people with different degrees of self-rated tinnitus severity.

## MATERIALS AND METHODS

### Participants

Data were obtained from 342 adults experiencing tinnitus who were recruited from National Institute for Health Research Nottingham Hearing Biomedical Research Unit (NIHR NHBRU) volunteer database, through advertisement of the study in the British Tinnitus Association’s (BTA) member magazine, *Quiet*, and through social media sites run by the BTA and the charity Hearing Link. Participants in the survey completed the TCQ as part of a battery of questionnaires online (n = 323; 94.4%) or in paper format (n = 19; 5.6%). Analysis of all the questionnaires in the survey is planned as a complete evaluation of the cognitive model of tinnitus distress (McKenna et al. 2014) using structural equation modeling. For a complete list of questionnaires used, please see Handscomb et al. (in press). Ethical approval was granted by the University of Nottingham’s School of Medicine research ethics committee (reference: G13022014 School of Medicine NIHR NHBRU Hearing). One participant completing the questionnaire on paper omitted one item on the positive subscale, and this individual was listwise deleted from the dataset. Otherwise, there were no missing data. The mean age of the sample was 55.0 years old (SD = 13.3 years), 185 (54.3%) were male and 138 (45.7%) were female. The mean duration of tinnitus was 14.0 years (SD = 13.7; range = 3 months to 69 years). All participants were stratified by their answer to the question: “How much of a problem is your tinnitus?” which was asked at the time of recruitment. This single item measure was used by Meikle et al. (2012) for scaling the global severity of tinnitus. Here, it was used to ensure that different degrees of tinnitus severity were represented in the study, which aimed to capture the experiences of people who are untroubled by tinnitus as well as those who are feeling distressed. To decide on an appropriate target number for each category, data from Zeman et al. (2012) were used. These researchers found that volunteers on the Tinnitus Research Initiative database proportionally fell into five categories, with more people reporting moderate levels of distress than either very low or very high levels. Using their figures as a guide, in this study, 35 participants (10.2%) were recruited in the “no problem” category, 85 (24.9%) in the “small problem” category, 102 (29.8%) in the “moderate problem category,” 83 (24.3%) in the “big problem” category, and 37 (10.8%) in the “very big problem” category. Recruitment to each category ended once the target sample size was reached. To test the validity of this form of categorization, mean scores on the Tinnitus Reaction Questionnaire (Wilson et al. 1991), a 26-item measure of tinnitus distress, were compared between problem categories. The maximum score on the TRQ is 104, with higher scores indicating greater tinnitus-related distress. Mean TRQ score was 2.23 (SD = 4.07) for the “no problem” group, 8.82 (SD = 9.26) for the “small problem” group, 23.20 (SD = 14.98) for the “moderate problem” group, 41.55 (SD = 21.91) for the “big problem group, and 66.35 (SD = 23.00) for the “very big problem” group. One-way analysis of variance (ANOVA) found differences in mean scores to be statistically significant between all problem categories [*F*(4,337) = 119.24, *p*< 0.001]. When the same problem scale was used by Meikle et al. (2012) during the development of the Tinnitus Functional Index (TFI)—a measure of global tinnitus distress—they found the differences between mean scores on the TFI to be large and statistically significant between each of the five problem categories in the expected direction (higher for “a big problem” than for “a moderate problem” and so on.) In the present study, the relationship between TRQ scores and problem scale ratings was investigated using Kendall’s *τ*. There was a strong, positive correlation between the two variables, *r* = 0.653, *p* < 0.001.

### Questionnaire

The TCQ (Wilson & Henry 1998) consists of a series of statements preceded by the words “I think” or “I tell myself,” for example, I think “if only the noise would go away.” The first 13 items refer to negative thoughts and the second 13 items refer to positive thoughts, for example, I think “there are things in life worse than tinnitus.” Participants are asked to indicate how often they have been aware of thinking each thought while noticing tinnitus. Responses are marked on a five-point Likert scale offering options of “never” = 0, “rarely” = 1, “occasionally” = 2, “frequently” = 3, or “very frequently” = 4. Positive items are reverse scored, so a high score on the positive subscale indicates a low level of positive thinking. The minimum total score is 0 and the maximum total score is 104. A score approximately halfway between 0 and 104 could indicate a low level of both positive and negative thinking, or the reverse, or a mixture of both components. Validation of the TCQ was carried out with 200 participants, 60 of whom were tinnitus clinic patients and the remainder were volunteers with tinnitus from the general population (Wilson & Henry 1998). The questionnaire was found to have very high internal consistency (*α* = 0.91) and adequate test–retest reliability (*r* = 0.88). Item–total correlations were between 0.43 and 0.66. It showed moderate correlation with measures of tinnitus distress. Correlations with mental health scales were weaker. This implies the TCQ measures a separate construct from overall tinnitus distress and from overall emotional disturbance. Principal components analysis revealed a two-factor solution, with items 1 to 13 forming a negative factor and items 14 to 26 forming a positive factor. The authors found no significant correlation between the two factors (*r* = 0.09) suggesting that the absence of positive thoughts does not necessarily imply the presence of negative thoughts.

### Analysis

Factor analysis was carried out using Mplus (version 7, Muthén and Muthén). A confirmatory factor model was specified and estimated using the robust weighted least squares estimator accounting for the categorical nature of response data. The first factor loading in each congeneric set was fixed by default at 1.0, and both means and variances of each variable were estimated. Model fit was based on a consensus of fit criteria and theoretical considerations. The fit criteria include the root mean square error of approximation (Steiger 1990) and 90% confidence intervals. A value of <0.05 was taken to represent good fit, errors of approximation of up to 0.08 were considered an acceptable absolute fit (Joreskog & Sorbom 1993), and a root mean square error of approximation of between 0.08 and 0.1 was considered a mediocre fit (MacCallum et al. 1996). Other fit indices included the Chi-square statistic, with a nonsignificant Chi-square representing a well-fitting model—although it may be affected by large sample sizes (Byrne 2012). The Comparative Fit Index (Bentler 1990) and Tucker Lewis Index (Tucker & Lewis 1973) are both comparative fit indices. For both, a value of >0.95 indicates a good fit (Hu & Bentler 1999).

The model tested was the two-factor model specified by Wilson and Henry (1998) with items 1 to 13 loading onto the negative factor and items 14 to 26 loading onto the positive factor. Models with and without correlated factors were estimated to assess whether there was a significant correlation between the two factors. Given the presence of an existing structure, CFA is the most appropriate initial approach; should the measurement proposed by Wilson and Henry not hold, we planned to use exploratory factor analysis (reflecting the categorical measurement of the variables).

In addition, a one-way ANOVA was carried out in SPSS (version 22) to investigate differences in mean scores on the TCQ between the five tinnitus problem categories

## RESULTS

### Demographics

There were significantly more males than females in the moderate problem group (χ^2^ = 0.59, *p* = 0.029). Otherwise, there were no significant differences in numbers of males and females in each group.

A one-way between groups ANOVA was conducted to explore the effect of age and problem category on tinnitus duration. There was no statistically significant difference in age for the five tinnitus problem categories [*F*(4,337) = 1.42, *p* = 0.23]. There was a statistically significant difference in mean tinnitus duration for the five problem categories [*F*(4, 332) = 2.89, *p* = 0.02]. Post-hoc comparisons indicated that the only significant between-groups difference was between the “no problem” group (mean duration = 19.5 years, SD = 15.03) and “very big problem” group (mean duration = 9.0 years, SD = 13.3).

### Reliability

Reliability was assessed using Cronbach’s *α*. The full questionnaire had high internal consistency (*α* = 0.901). Internal consistency was also high for both negative and positive subscales (*α* = 0.959 and 0.929, respectively.)

### Confirmatory Factor Analysis

Initial testing of the two-factor structure of the TCQ allowing for the two factors to correlate resulted in a poor fit (Table 1). The two factors were found to be uncorrelated (*ρ* = −0.03). The second CFA that fixed the correlation between the two variables to be zero resulted in a much better fit. Fit indices for both models tested are shown in Table 1. Factor loadings for all items in the better fitting model (model 2) were high, positive, and statistically significant (Table 2).

**TABLE 1:**
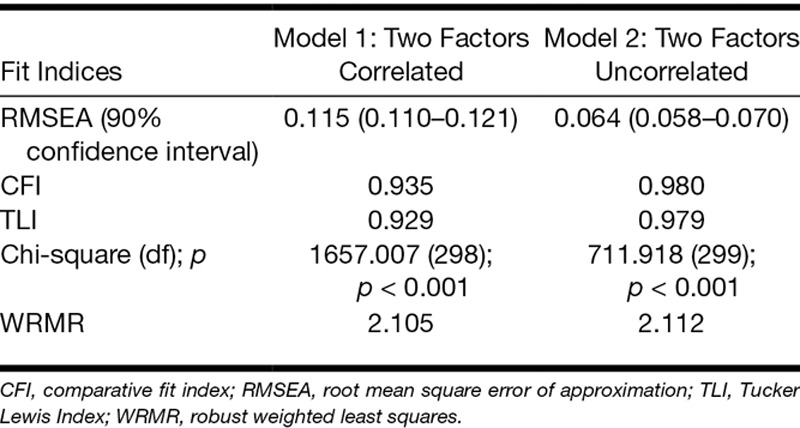
Fit indices for two alternative models of the Tinnitus Cognitions Questionnaire with two factors correlated and uncorrelated

**TABLE 2:**
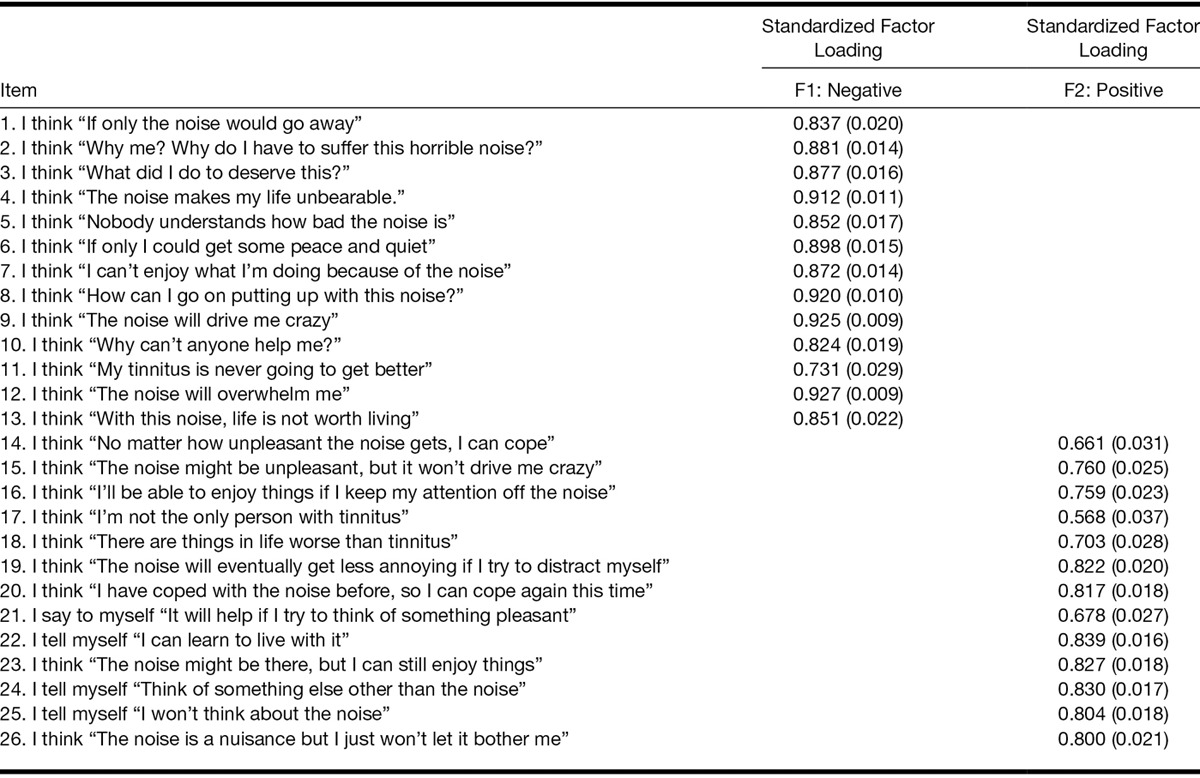
Standardized factor loadings (SEs) for a two-factor model of the Tinnitus Cognitions Questionnaire with correlation between factors fixed at 0

### Individual Items

Mean scores across the complete dataset for each TCQ item were calculated. The items with the highest mean scores were items: 1, “if only the noise would go away” (M = 2.60, SD = 1.20); 11, “my tinnitus is never going to get better” (M = 2.56, SD = 1.32); and 6, “if only I could get some peace and quiet” (M = 2.39, SD = 1.31). The lowest mean scores were obtained by items: 12, “the noise will overwhelm me” (M = 1.32, SD = 1.24); 3, “what did I do to deserve this?” (M = 1.03, SD = 1.28); and 13, “with this noise, life is not worth living” (M = 0.58, SD = 1.02).

### Mean TCQ (Sub)scale Scores

The overall mean TCQ score was 43.86 (SD = 17.20). Mean scores for the total TCQ and for the two subscales according to “problem category” are shown in Table 3.

**TABLE 3:**
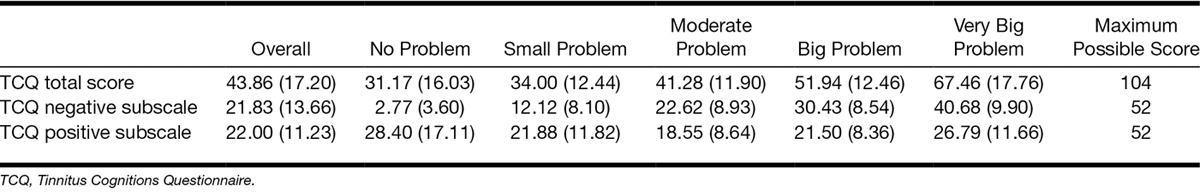
Mean TCQ scores (SD) according to tinnitus problem category

A one-way between groups ANOVA was conducted to explore the effect of problem category on mean score on the TCQ using Bonferroni correction for multiple comparisons. For the negative subscale, there was a statistically significant difference across the five problem categories [*F*(4,337) = 143.58, *p* < 0.001]. In fact, post-hoc comparisons using the Tamhane T2 test indicated that the differences between mean scores were statistically significant (*p* < 0.05; corrected) for all problem groups.

For the positive subscale, there was a statistically significant difference across the five problem categories [*F*(4,336) = 7.47, *p* < 0.001]. However, post-hoc comparisons using the Tamhane T2 test indicated that the differences between mean positive scores were only statistically significant for the “no problem” group (M = 28.40, SD = 17.11) compared with the “moderate problem” group (M = 18.55, SD = 8.64, *p* = 0.02) and for the “moderate problem” group compared with the “very big problem” group (M = 26.79, SD = 11.66, *p* = 0.002).

Overall, there was a statistically significant difference in total TCQ scores for the five problem categories [*F*(4,336) = 7.47, *p* < 0.001]. Post-hoc comparisons using the Tamhane T2 test indicated that the mean total score for the “no problem” group (M = 31.17, SD = 16.03) was not significantly different from the mean total score for the “small problem” group (M = 34.00, SD = 12.44, *p* = 0.99). Mean scores for all other groups were significantly different (*p* < 0.05; corrected).

## DISCUSSION

The findings of this study have contributed to knowledge about the type and frequency of thoughts people have relative to the severity of their tinnitus, as well as providing support for the use of the TCQ in tinnitus research and clinical practice.

The TCQ has previously only been validated in 200 Australian individuals, some of whom were clinic patients and some of whom were volunteers (Wilson & Henry 1998). Our findings, which came from a somewhat larger UK-based nonclinical population, were broadly similar to those of the original study. The overall mean TCQ score was equivalent to that of Wilson and Henry’s mixed population (M = 47.16; SD = 12.71). The same three items (1, 6, and 11) had the highest mean scores, suggesting frequently wishing to be free of the noise, longing for peace, and quiet but despairing of this possibility seem to be common ways of thinking. Few people in either study reported thinking that “life is not worth living” with tinnitus (item 13); one might expect this item only to apply to those experiencing the very greatest distress. Both studies found the positive and negative subscales to be uncorrelated. Factor analysis also found the proposed two-factor structure to have good psychometric properties in this population. Very high internal consistency suggests that there may be some redundant items and the TCQ could potentially be reduced to a smaller number of items.

The significantly higher scores on the whole questionnaire and particularly on the negative subscale for people who rated their tinnitus as more of a problem supports the idea that more negative thinking is associated with a worse experience of tinnitus in this nonclinical population. It is interesting to note that, on the positive subscale, scores were not higher when self-rated tinnitus severity was higher. Rather, the highest mean score on the positive subscale (indicating fewer or less frequent positive thoughts) was obtained by the “no problem” group. Many people in this group indicated that they “never” or “rarely” had such thoughts as “the noise might be there, but I can still enjoy things” or “I have coped with the noise before, so I can cope again this time.” It seems likely that many people who do not find their tinnitus to be a problem feel no need to engage in positive thinking. They simply do not think about their tinnitus very much at all. One of the survey respondents who rated tinnitus as “not a problem” also commented: “the…[TCQ] questionnaire I found quite difficult as I just don’t think about these things, I felt that my responses might come over as negative when actually I just do not have these thoughts about tinnitus at all.” Similar conclusions have been drawn from research into the concept of tinnitus acceptance, which can be defined as “an open, mindful and nonevaluative approach to internal experiences” (Hesser et al. 2012, p. 650). In questionnaire studies, both Hesser et al. (2015) and Riedl et al. (2015) found tinnitus acceptance to be negatively correlated with tinnitus distress. In other words, people who were not very distressed by their tinnitus tended not to become engaged in thinking about it.

In the present study, the lowest mean score on the positive subscale (indicating a greater number of and/or more frequent positive thoughts) was obtained by the “moderate problem” group, while the “very big problem” group scored relatively high on this subscale. It may be that positive thinking is a strategy deliberately used by people to better cope with troubling tinnitus or that people who class tinnitus as a “moderate problem” switch between negative and positive thinking, perhaps depending on mood or environment. It is likely that those people who class tinnitus as a “very big problem” are simply unable to engage in positive thinking most of the time, although some positive thinking is still occurring in this group. These observations and the lack of correlation between positive and negative subscales suggest that positive thoughts do not “cancel out” negative ones. Similar findings were reported by Budd and Pugh (1996), who found that “effective coping” was not associated with lower distress, whereas “lack of maladaptive coping” was. The indication here is that positive thinking is not characteristic of nonbothersome tinnitus, whereas lack of negative thinking is.

The findings of the present study have some important implications for therapy. Here, we found that people who rate their tinnitus as a “moderate problem” are able to think positively about it at least some of the time. This is something that can be built on during counseling. People with very problematic tinnitus may need more intensive help to change their thinking. It is notable that Conrad et al. (2015a) found that participants who engaged in the most catastrophic thinking had poorer outcomes than others after clinician-guided internet-based CBT but not after face-to-face CBT. Here, face-to-face therapy allowed for more intensive therapeutic discussion, possibly explaining the difference. From any starting point, becoming a member of the “not a problem” group is clearly the most desirable outcome of therapy. To be like members of this group, our findings indicate that people need to learn to stop thinking about their tinnitus altogether. Deliberately replacing negative thoughts with more positive ones may or may not be part of this transition. Significant reductions in tinnitus-related emotional distress have been demonstrated in several studies, which involve cognitive restructuring exercises (Henry & Wilson 1996; Hiller & Haerkotter 2005; Cima et al. 2012; Conrad et al. 2015a), but it is unclear how much of a contribution such exercises make to overall improvement above other things such as attention-shifting or behavior change. It is also as yet unknown whether still greater improvements might come about through mindful meditation, during which people learn to disengage with their thoughts altogether (Williams et al. 2007). Results of ongoing trials may help to answer how such psychological therapies bring about patient benefit.

Overall, the TCQ appears to be a reliable measure of positive and negative thinking about tinnitus with good psychometric properties in our study population. Its internal consistency is notably high, suggesting that some items may be redundant. Future research might look at reducing the scale to a smaller set of key items. Nevertheless, we recommend use of the TCQ for a number of reasons. First, negative thinking is seen to be crucial to the development of tinnitus distress (McKenna et al. 2014), and yet, little data have been collected about the content and frequency of thoughts related to tinnitus. Widespread use of a measure such as the TCQ would help build an evidence set of thinking patterns amongst different groups of people with tinnitus to refine theory. Second, current psychological interventions for tinnitus, particularly CBT, ACT, and mindfulness, have contrasting ideas about how best to manage thoughts. Further investigation of whether, how, and to what degree different forms of therapy affect both negative and positive thinking would be helpful in modifying psychological approaches for greater benefit. Including the TCQ as a secondary outcome measure in clinical trials of psychological intervention for tinnitus would be one way of gathering information on this issue.

A limitation of the present study is that only people from the general population were tested. Validation of the TCQ using clinical populations is an important next step. A limitation of the TCQ itself is that the thoughts it lists, although derived from patient interviews, it do not appear to have been selected through a systematic process. Other measures of tinnitus-related thinking are adapted from pain cognition scales (Flor & Schwarz 2003; Cima et al. 2011) and were not developed with tinnitus patients in mind. We do not yet have sufficient knowledge of how people with tinnitus think about it. Surveys and qualitative interview studies would help to build a more accurate picture.

## CONCLUSIONS

Negative thinking appears to be linked to a worse experience of tinnitus, while positive thinking does not appear to be associated with nontroublesome tinnitus. Further investigation of this finding in a clinical population would be informative, for example by calculating scores on positive and negative subscales and comparing them across groups of help-seekers and non help-seekers.

The TCQ is a unique measure of positive and negative thinking related to tinnitus, and its use should be considered by researchers and clinicians offering psychological therapies.

## ACKNOWLEDGMENTS

L.E.H. is funded by the British Tinnitus Association. D. A. H. and D. J. H. are funded by *NIHR* Biomedical Research Unit program; however, the views expressed in this article are those of the authors and not necessarily those of the *NIHR*, the *NHS*, or the Department of Health.

## ANNOUNCEMENT

### WORLD HEARING DAY: 3 MARCH 2017

#### ACTION FOR HEARING LOSS: MAKE A SOUND INVESTMENT

The World Hearing Day is an annual advocacy event, coordinated by the World Health Organization (WHO). It is observed on 3rd March each year and aims to raise awareness on hearing loss and promote actions for ear and hearing care across the world. The day is marked by a different theme each year and accompanied by evidence-based advocacy tools and messages, which are released by WHO. A large number of individuals, organizations and governments have joined this effort in order to advocate for prioritization of hearing care.

The theme for 2017 is ‘Action for hearing loss: make a sound investment’. This will focus on the economic aspects of hearing loss and cost effectiveness of interventions to reduce its prevalence and impact.

Information products and advocacy materials will be made available by WHO on its webpage http://www.who.int/pbd/deafness/world-hearing-day/en/. WHO invites all stakeholders in the field of ear and hearing care to undertake activities to mark this day and join this global initiative.

## References

[R1] Bentler P. M Comparative fit indexes in structural models. Psychol Bull(1990)107 238 246 2320703 10.1037/0033-2909.107.2.238

[R2] Budd R. J. Pugh R Tinnitus coping style and its relationship to tinnitus severity and emotional distress. J Psychosom Res(1996)41 327 335 8971662 10.1016/s0022-3999(96)00171-7

[R3] Byrne B Structural Equation Modelling with MPlus(2012)Hove, England Routledge

[R4] Cima R. F. F. Crombez G. Vlaeyen J. W. S Catastrophizing and fear of tinnitus predict quality of life in patients with chronic tinnitus. Ear Hear(2011)32 634 641 21399500 10.1097/AUD.0b013e31821106dd

[R5] Cima R. F. F. Maes I. H. Joore M. A. Specialised treatment based on cognitive behaviour therapy versus usual care for tinnitus: A randomised controlled trial. Lancet(2012)379 1951 1959 22633033 10.1016/S0140-6736(12)60469-3

[R6] Conrad I., Kleinstaeuber M., Jasper K. (2015a). The changeability and predictive value of dysfunctional cognitions in cognitive behavior therapy for chronic tinnitus.. Int J Behav Med.

[R7] Conrad I., Kleinstauber M., Jasper K. (2015b). The role of dysfunctional cognitions in patients with chronic tinnitus.. Ear Hear.

[R8] Davis A. El Refaie A Tyler R. Epidemiology of tinnitus. In Tinnitus Handbook(2000)San Diego, CA Thomson Learning; 1 23.

[R9] Flor H. Schwarz M Schwarz M. Andrasik F. Tinnitus: Nothing is as loud as a sound you are trying not to hear. In Biofeedback: A Practitioner’s Guide(2003)New York, NY Guildford

[R10] Fowler E The illusion of loudness of tinnitus - Its etiology and treatment. Laryngoscope(1942)275 285 June

[R11] Gans J. J. O’Sullivan P. Bircheff V Mindfulness based tinnitus stress reduction pilot study: A symptom perception-shift program. Mindfulness(2014)5 322 333

[R12] Hallam R. S. Rachman S. Hinchcliffe R Rachman S. Psychological aspects of tinnitus. In Contributions to Medical Psychology(1984)Oxford, UK Pergamon

[R13] Handscomb L., Hall D. A., Shorter G (in press). Online data collection to evaluate a theoretical cognitive model of tinnitus.. Am J Audiol.

[R14] Hayes S. C. Villatte M. Levin M. Open, aware, and active: Contextual approaches as an emerging trend in the behavioral and cognitive therapies. Ann Rev Clin Psychol(2011)7 141 168 21219193 10.1146/annurev-clinpsy-032210-104449

[R15] Henry J. Wilson P The psychological management of tinnitus: Comparison of a combined cognitive educational program, education alone and a waiting list control. Int Tinnitus J(1996)2 9 20 10753339

[R16] Hesser H. Weise C. Westin V. Z. A systematic review and meta-analysis of randomized controlled trials of cognitive-behavioral therapy for tinnitus distress. Clin Psychol Rev(2011)31 545 553 21237544 10.1016/j.cpr.2010.12.006

[R17] Hesser H. Gustafsson T. Lunden C. A randomized controlled trial of internet-delivered cognitive behavior therapy and acceptance and commitment therapy in the treatment of tinnitus. J Consult Clin Psychol(2012)80 649 661 22250855 10.1037/a0027021

[R18] Hesser H. Westin V. Z. Andersson G Acceptance as a mediator in internet-delivered acceptance and commitment therapy and cognitive behavior therapy for tinnitus. J Behav Med(2014)37 756 767 23881309 10.1007/s10865-013-9525-6

[R19] Hesser H. Bankestad E. Andersson G Acceptance of tinnitus as an independent correlate of tinnitus severity. Ear Hear(2015)36 e176 e182 25665072 10.1097/AUD.0000000000000148

[R20] Hiller W. Goebel G When tinnitus loudness and annoyance are discrepant: Audiological characteristics and psychological profile. Audiol Neurootol(2007)12 391 400 17664870 10.1159/000106482

[R21] Hiller W. Haerkotter C Does sound stimulation have additive effects on cognitive-behavioral treatment of chronic tinnitus? Behav Res Ther(2005)43 595 612 15865915 10.1016/j.brat.2004.03.012

[R22] Hoare D. J. Kowalkowski V. L. Kang S. J. Systematic review and meta-analyses of randomized controlled trials examining tinnitus management. Laryngoscope(2011)121 1555 1564 21671234 10.1002/lary.21825 PMC3477633

[R23] Hu L.-T. Bentler P. M Cutoff criteria for fit indexes in covariance structure analysis: Conventional criteria versus new alternatives. Struct Equ Model(1999)6 1 55

[R24] Joreskog K. Sorbom D Structural Equation Modelling with the SIMPLIS Command Language(1993)Chicago, IL Scientific Software, Inc.

[R25] Kroner-Herwig B. Frenzel A. Fritsche G. The management of chronic tinnitus - Comparison of an outpatient cognitive-behavioral group training to minimal-contact interventions. J Psychosom Res(2003)54 381 389 12670617 10.1016/s0022-3999(02)00400-2

[R26] MacCallum R. C. Browne M. W. Sugawara H. M Power analysis and determination of sample size for covariance structure modeling. Psychol Methods(1996)1 130 149

[R27] McKenna L. Handscomb L. Hoare D. A scientific cognitive behavioral model of tinnitus: Novel conceptualizations of tinnitus distress. Front Neurol(2014)5 1 15 25339938 10.3389/fneur.2014.00196 PMC4186305

[R28] Meikle M. B. Henry J. A. Griest S. E. The tinnitus functional index: Development of a new clinical measure for chronic, intrusive tinnitus (vol 33, pg 153, 2012). Ear Hear(2012)33 443 443 10.1097/AUD.0b013e31822f67c0 22156949

[R29] Philippot P. Nef F. Clauw L. A randomized controlled trial of mindfulness-based cognitive therapy for treating tinnitus. Clin Psychol Psychother(2012)19 411 419 21567655 10.1002/cpp.756

[R30] Riedl D. Rumpold G. Schmidt A. The influence of tinnitus acceptance on the quality of life and psychological distress in patients with chronic tinnitus. Noise Health(2015)17 374 381 26356381 10.4103/1463-1741.165068 PMC4900501

[R31] Sadlier M. Stephens S. D. G. Kennedy V Tinnitus rehabilitation: A mindfulness meditation cognitive behavioural therapy approach. J Laryngol Otol(2008)122 31 37 17451612 10.1017/S0022215107007438

[R32] Segal Z. Williams M. Teasdale J Mindfulness-Based Cognitive Therapy for Depression: A New Approach to Preventing Relapse(2002)New York, NY Guilford

[R33] Steiger J. H Structural model evaluation and modification: An interval estimation approach. Multivariate Behav Res(1990)25 173 180 26794479 10.1207/s15327906mbr2502_4

[R34] Tucker L. R. Lewis C Reliability coefficient for maximum likelihood factor analysis. Psychometrika(1973)38 1 10

[R35] Weise C. Hesser H. Andersson G. The role of catastrophizing in recent onset tinnitus: Its nature and association with tinnitus distress and medical utilization. Int J Audiol(2013)52 177 188 23301660 10.3109/14992027.2012.752111

[R36] Westin V. Z. Schulin M. Hesser H. Acceptance and commitment therapy versus tinnitus retraining therapy in the treatment of tinnitus: A randomised controlled trial. Behav Res Ther(2011)49 737 747 21864830 10.1016/j.brat.2011.08.001

[R37] Williams M. Teasdale J. Segal Z. The Mindful Way Through Depression(2007)London Guilford

[R38] Wilson P. Henry J Tinnitus cognitions questionnaire: Development and psychometric properties of a measure of dysfunctional cognitions associated with tinnitus. Int Tinnitus J(1998)4 22 30 10753381

[R39] Wilson P. H. Henry J. Bowen M. Tinnitus reaction questionnaire - Psychometric properties of a measure of distress associated with tinnitus. J Speech Hear Res(1991)34 197 201 2008074

[R40] Zeman F. Koller M. Schecklmann M. Tinnitus assessment by means of standardized self-report questionnaires: Psychometric properties of the Tinnitus Questionnaire (TQ), the Tinnitus Handicap Inventory (THI), and their short versions in an international and multi-lingual sample. Health Qual Life Outcomes(2012)10 10.1186/1477-7525-10-128 PMC3541124 23078754

[R41] Zenner H. P. Zalaman I. M Cognitive tinnitus sensitization: Behavioral and neurophysiological aspects of tinnitus centralization. Acta Oto-Laryngol(2004)124 436 439 10.1080/00016480410016333 15224870

